# Supplementary material for: Regulatory sequence-based discovery of anti-defense genes in archaeal viruses
Source: Nat Commun. 2024 May 2;15:3699. doi: 10.1038/s41467-024-48074-x (PMC11065993; doi:10.1038/s41467-024-48074-x)
Supplement: Supplementary file 1 — Supplementary information file [file 41467_2024_48074_MOESM1_ESM.pdf]

Supplementary information

Regulatory sequence-based discovery of anti-defense genes in archaeal viruses

Yuvaraj Bhoobalan-Chitty[1,3,*], Shuanshuan Xu[1,3], Laura Martinez-Alvarez[1,3], Svetlana Karamycheva[2], Kira S. Makarova[2], Eugene V. Koonin[2], Xu Peng[1,*]

[1]Department of Biology, University of Copenhagen, Copenhagen N, Denmark

[2]National Center for Biotechnology Information, National Library of Medicine, NIH, Bethesda, MD, USA.

[3]These authors contributed equally to this work.

*Address correspondence to Xu Peng, peng@bio.ku.dk or Yuvaraj Bhoobalan-Chitty, yuvarajb@bio.ku.dk

Supplementary figures

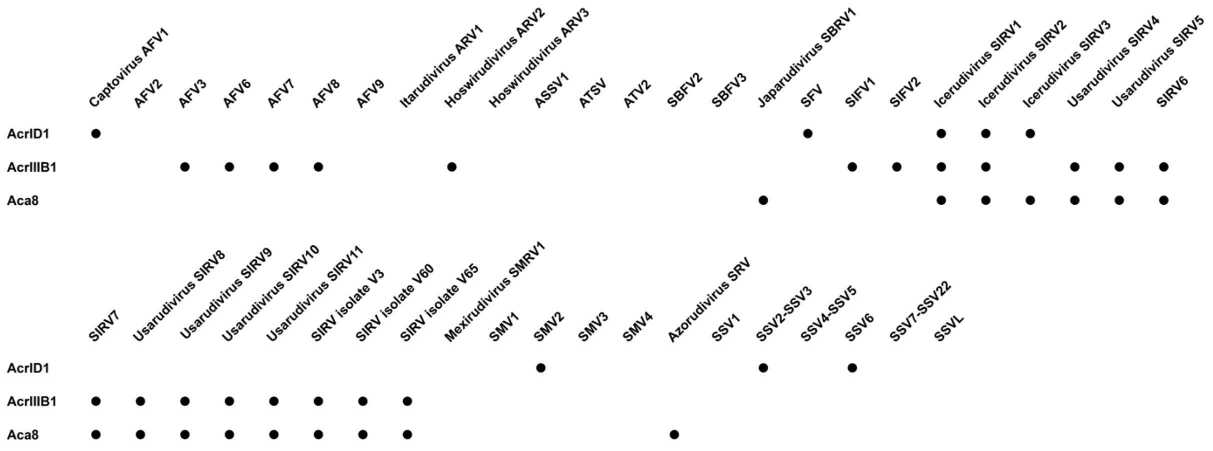

Supplementary figure 1. Distribution of AcrID1, AcrIIIB1 and Aca8 among Archaeal viruses. Black dots indicate a presence of AcrID1, AcrIIIB1 or Aca8 coding sequences in the respective virus genome.

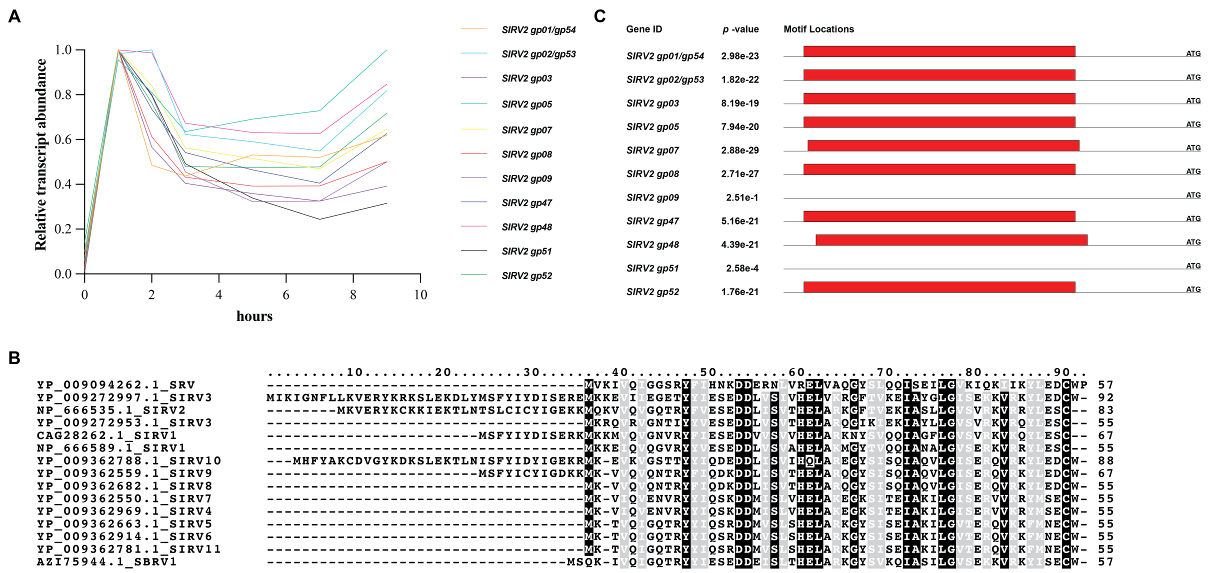

Supplementary figure 2. Existence of regulatory sequences upstream of early SIRV2 genes. A. Expression pattern of SIRV2 early genes at 0 – 9 hours post infection in *S. islandicus* LAL14/1 (adapted from Quax et al., 2013), the highest number of transcripts of an individual gene during the 10 hours is normalized to 1 and used as the maximum value to estimate fractions at other times points. **B.** Multiple sequence alignment of gp01 (Aca8) homologs found among archaeal viruses. **C.** MEME-RSAT identified motif distribution among *SIRV2* early genes.

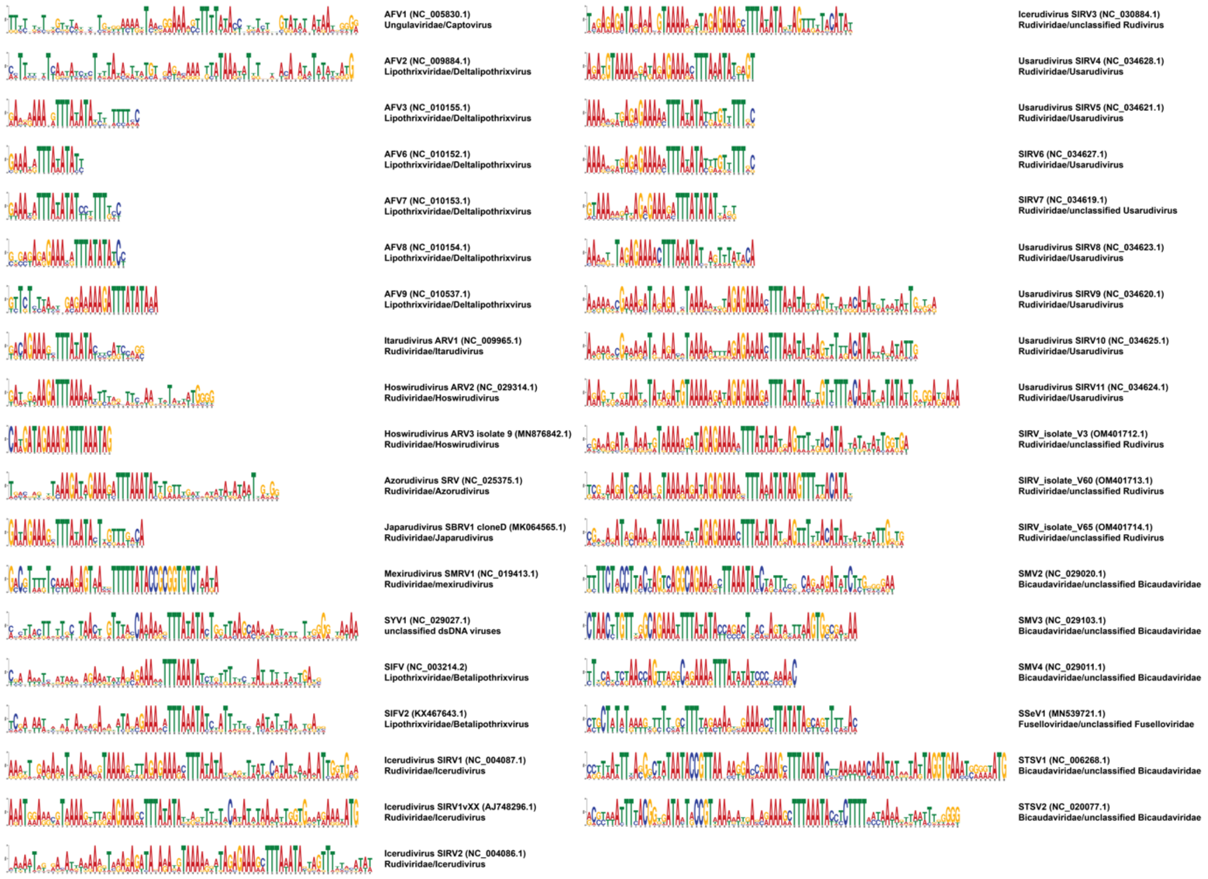

Supplementary figure 3. ADG sequence motifs. Motifs from individual viruses as determined by MEME-suite used in the identification of ADG.

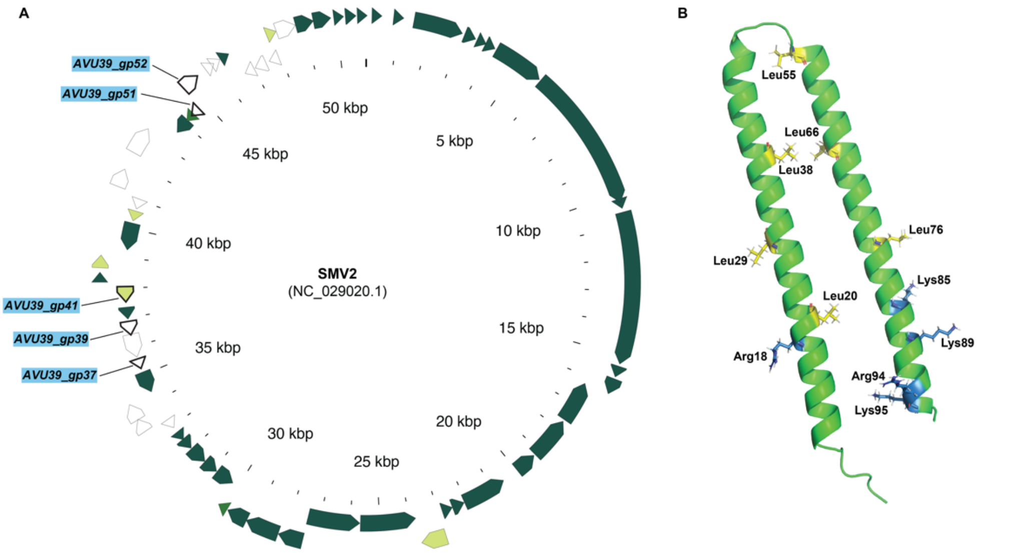

Supplementary figure 4. ADGs in SMV2. A. MEME-RSAT prediction of potential ADGs in SMV2 are highlighted with labels and black lines. Proteins conserved (established using BLAST) among SMV1-4 are shown in dark green, light green arrows indicate genes shared between 2 to 3 *SMVs,* and empty arrows represent genes exclusively found in SMV2. **B.**

AlphaFold predicted structure of SMV2 gp37. Regularly interspaced leucine residues conserved between AVU39_gp37 (SMV2) and L7H13_norf1 are shown in yellow. Conserved basic residues are shown in light blue.

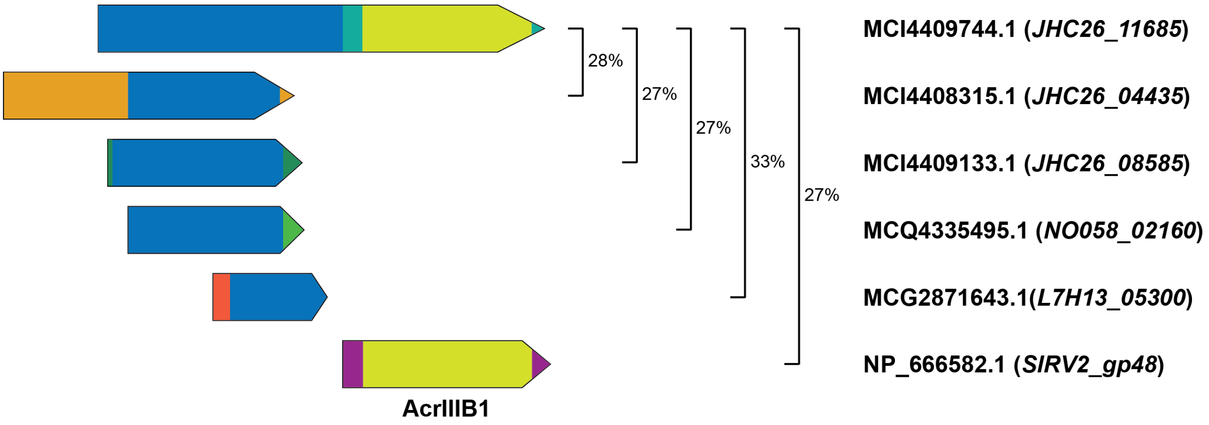

Supplementary figure 5. MCI14409744.1 a multifunctional anti-defense protein. Comparison of MCI4409744.1 (JHC26_11685) with homologs, showing JHC26_11685 to be a fusion protein of multiple proteins including AcrIIIB1 and an unknown putative anti-defense element (N-Terminal) conserved among other contigs. The numbers represent percentage identity of the homologs in comparison to JHC26_11685.

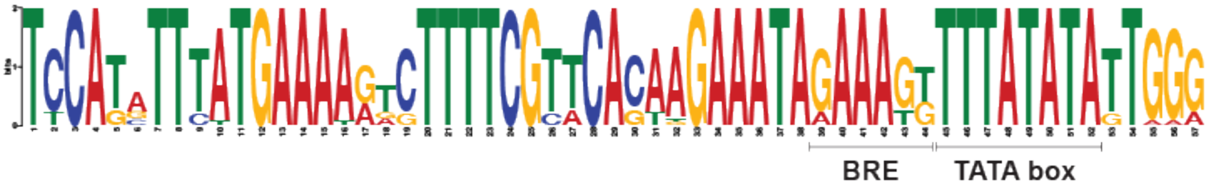

Supplementary figure 6. Conservation of BRE and TATA-box among the promoter sequences of CRISPR-Cas subtype I-A interference gene cluster in *Sulfolobus* strains.

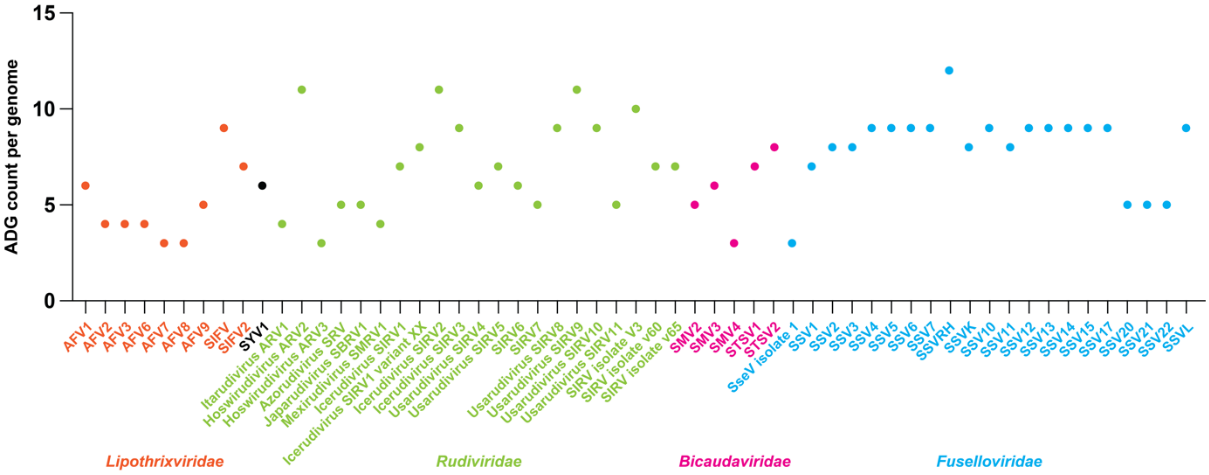

Supplementary figure 7. ADG counts in viral genomes. The number of ADGs of individual virus analyzed in this study is shown. Virus families and their respective members are differentiated based on color, *Lipothrixviridae* (orange), *Rudiviridae* (green), *Bicaudaviridae* (pink) and *Fuselloviridae* (blue). The virus *SYV1*, shown in black is unclassified.

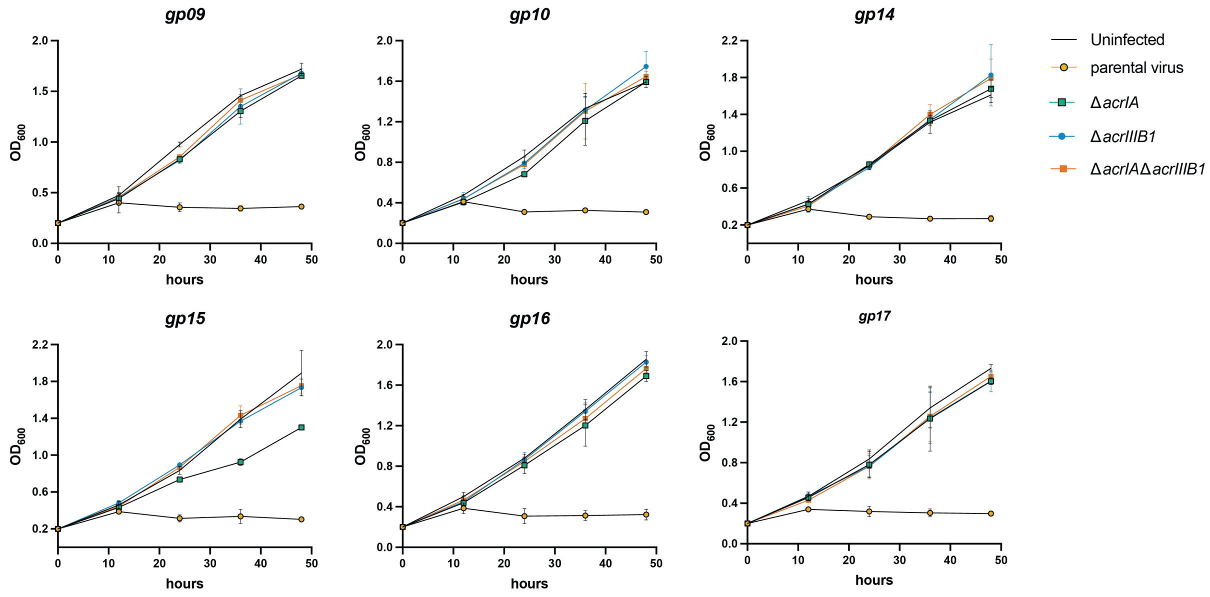

Supplementary figure 8. Screening for CRISPR-Cas subtype I-A inhibitors among predicted SIFV2 ADGs. Growth curves of *S. islandicus* LAL14/1 Δ*cas6(I-D)* carrying either an empty plasmid or a plasmid encoding SIFV2 *gp09, gp10, gp14, gp15, gp16* and *gp17*. Cultures were uninfected or infected with parental virus (SIRV2M), Δ*acrIA* (SIRV2MΔ*gp45-gp47*), Δ*acrIIIB1* (SIRV2MΔ*gp48*) and Δ*acrIA*Δ*acrIIIB1* (SIRV2MΔ*gp45-gp48*). Results from two biological replicates are shown, as mean ± SD. Source data are provided below (Supplementary table 2).

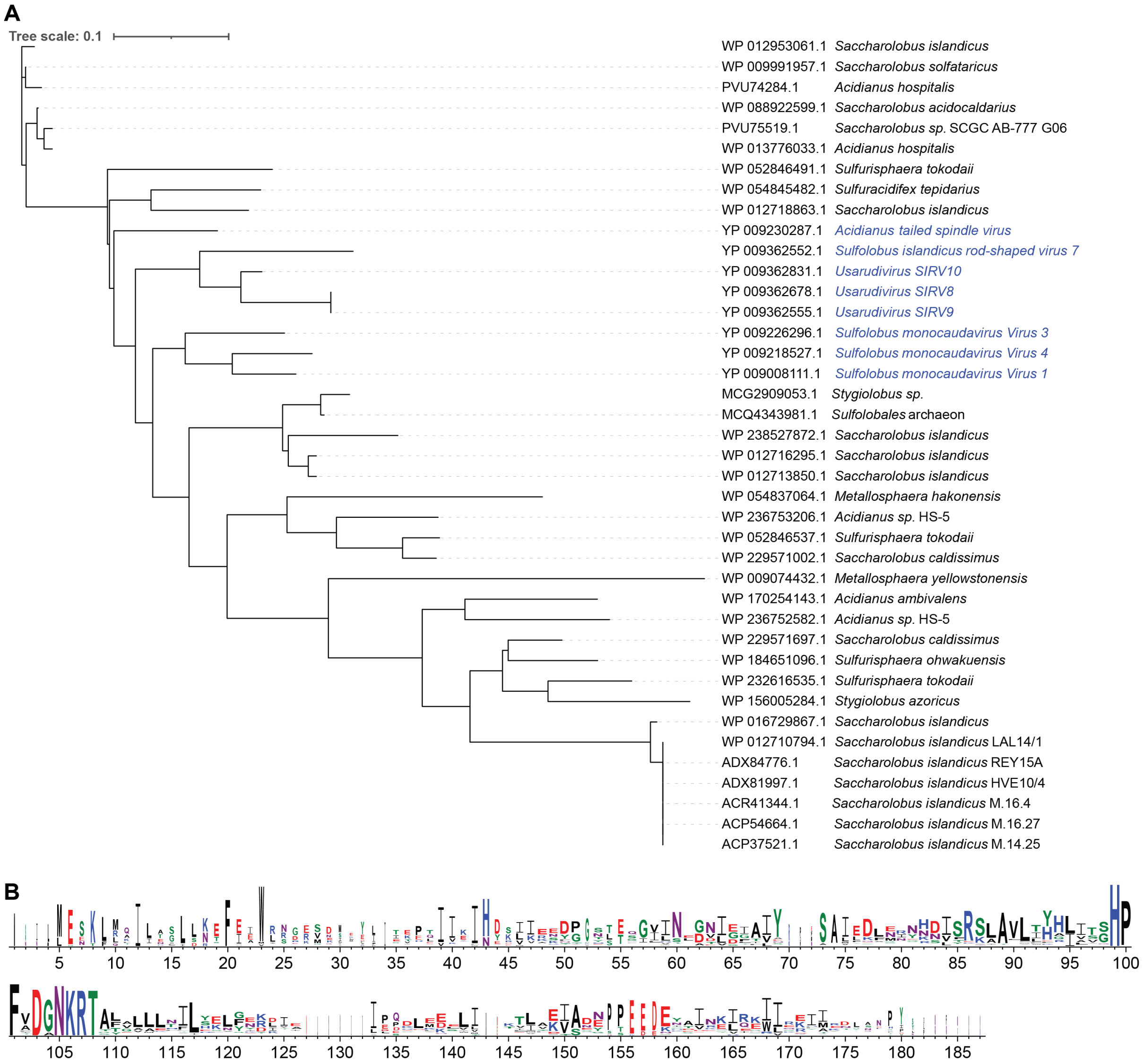

Supplementary figure 9. Phylogenetic analysis of ADG.17 (antitoxin) and MSA of SIL_0731 (toxin). A. Phylogenetic tree of the host (black) and virally (blue) encoded Phd/Doc antitoxin homologs. **B.** Weblogo of SIL_0731 multiple sequence alignment illustrating the conservation of the active site (HXFX(D/E)(A/G)N(G/K)R) withing the Doc toxin. Multiple sequence alignments are provided in supplementary file 3.

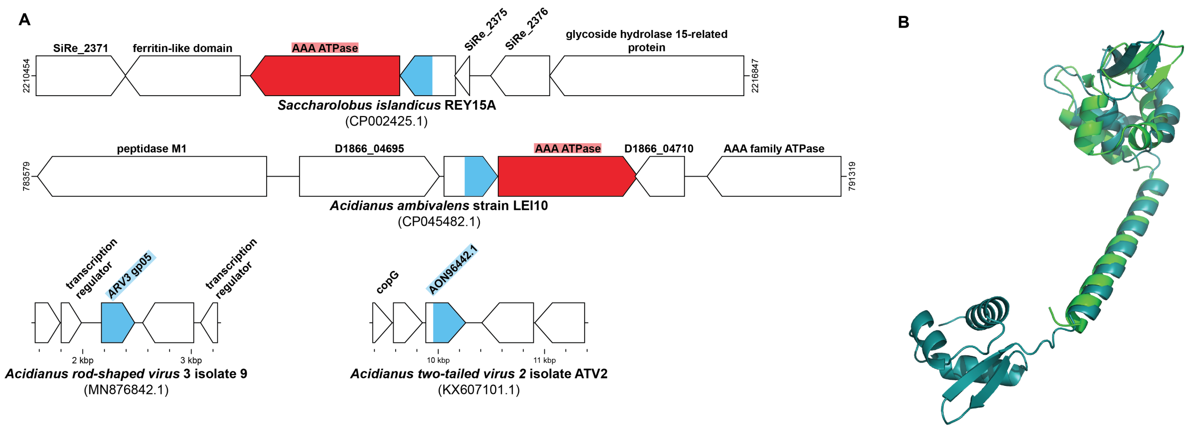

Supplementary figure 10. A. Illustration of a possible two-component defense system conserved among Sulfolobales as represented here in *S. islandicus* REY15A and *Acidianus ambivalens* LEI 10. Viral homologs of the likely antitoxin (repressor of the toxin) in ARV3 and ATV2. **B.** Overlay of AlphaFold predicted structures of ARV3 gp05 (green) and SiRe_2374 (deepteal).

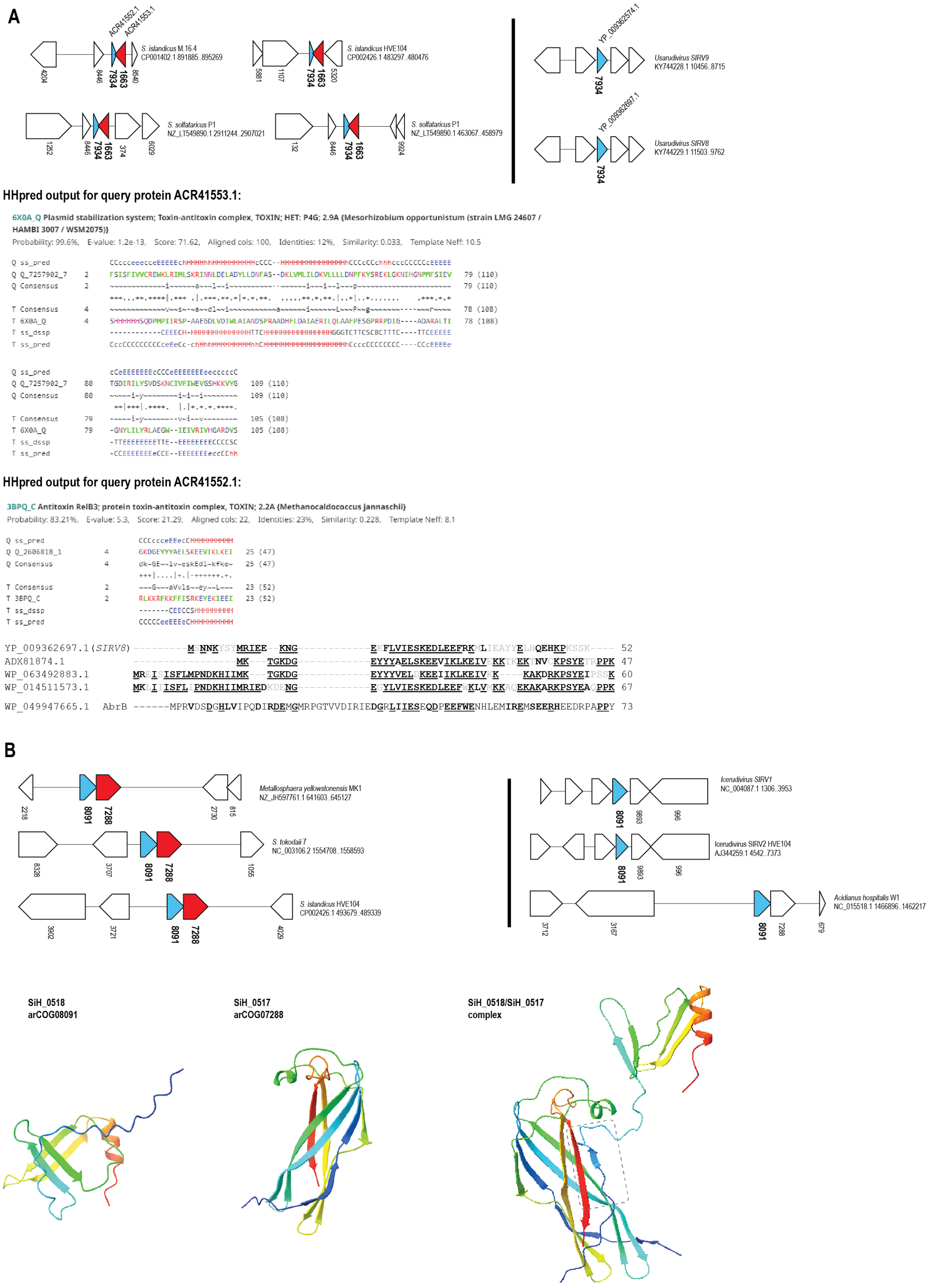

Supplementary figure 11. Examples of putative antitoxins encoded in viral genomes. **A.** AbrB-RelE toxin-antitoxin system. Genes are represented by arrows. Species name, nucleotide accession and coordinates of the respective locus are indicated on the right. Host loci are shown

on the left side and separated from viral loci by the black line. arCOG numbers are indicated under each arrow. Arrows corresponding to toxins are colored red and to antitoxin – blue. HHpred output demonstrating sequence similarity of arCOG01663 and arCOG07934 proteins to RelE and AbrB, respectively, is shown in the middle of the panel. Alignment of host and viral AbrB proteins with a representative AbrB protein is shown at the bottom of the panel. **B.** arCOG08091- arCOG07288 putative toxin-antitoxin systems. Designations are the same as in panel A. Alphafold2 predicted structures of the protein representatives of arCOG08091, arCOG07288 and the complex of these proteins are shown underneath the schemes. Dashed rectangle shows the area of interaction between the predicted toxin and antitoxin.

Supplementary table

Supplementary table 1: Oligonucleotides used in this study

Plasmid construct	Oligonucleotide	Sequence (5' -> 3')
p*gp09*$_{araS2}$	SIFV2gp09 NdeI For	ATTCATATGGCAAAACAAGAAGTGAAACAA
	SIFV2gp09 NotI Rev	ATTGCGGCCGCTTATTCAAGTAATATTTTCTTTTTTCTGT
p*gp10*$_{araS2}$	SIFV2gp10 NdeI For	ATTCATATGGTAAGTTCAAAAGATAAAATAAAAG
	SIFV2gp10 NotI Rev	ATTGCGGCCGCTTACCCCTTTTTCGTTTTCGTTAA
p*gp14*$_{araS2}$	SIFV2gp14 NdeI For	ATTCATATGGGTGGGAAAACATTAATTATAAA
	SIFV2gp14 NotI Rev	ATTGCGGCCGCTTATTCTTCTGCAAAAGACACTTC
p*gp15*$_{araS2}$ / p*gp15*$_{araS\text{ - SD}}$	SIFV2gp15 NdeI For	ATTCATATGGTGGAAGTAAAACAAAAAACC
	SIFV2gp15 NotI Rev	ATTGCGGCCGCTTACTCAATTTCGATTTCTGCAT
p*gp16*$_{araS2}$	SIFV2gp16 NdeI For	ATTCATATGAATGAACAGATAGAATTTGG
	SIFV2gp16 NotI Rev	ATTGCGGCCGCTTATCTTTCATAATTAGAATATATAAGATC
p*gp17*$_{araS2}$	SIFV2gp17 NdeI For	ATTCATATGGCGGAAATAACATCCAAAAAG
	SIFV2gp17 NotI Rev	ATTGCGGCCGCTTAATCAACTTCTATCATTAGTCC
p*SiL_0730*	SiL_0730 NdeI For	GGAATTCCATATGTATAAGAGAAAAATGACTGAA
	SiL_0730 NotI Rev	AAAAAGCGGCCGCCTAATCCTTATTAGAATCACGT
p*SiL_0731*	SiL_0731 NdeI For	GGAATTCCATATGGAAAGTAAGTTGAGAGCCA
	SiL_0731 NotI Rev	AAAAAGCGGCCGCCTAATAAGGATTAGCTAAATTTCTC
pSMV*1gp44*	SMV1gp44 NdeI For	GGAATTCCATATGGTGGCAACGCATAAATTAA
	SMV1gp44 NotI Rev	AAAAAGCGGCCGCTCACCCCTGGTTTTTATTCTC
p*SiL_0730**/ *SiL_0731*	SiL_0730Mut For	AAAAATGACTTGACAAGTTTCCGAGATTCAGAA
	SiL_0730Mut Rev	GGAAACTTGTCAAGTCATTTTTCTCTTATACATATG

pSMV1*gp44**/ *SiL_0731*	SMV1gp44Mut For	GCATAAATGAACAGATGAGCAGAAAAAGATC
	SMV1gp44Mut Rev	CATCTGTTCATTTATGCGTTGCCACCATAT
p*SiL_0730*/ *SiL_0731** or pSMV1*gp44*/ *SiL_0731**	SiL_0731Mut For	GTAAGTTGTGAGCCATCATCACCTCACTT
	SiL_0731Mut Rev	GATGGCTCACAACTTACTTTCCATATGCTCG
pET30a(+)/ *SiL_0730chis*	SiL_0730chis For	GAAATAATTTTGTTTAACTTTAAGAAGGAGATATACATATG TATAAGAGAAAAATGACTG
	SiL_0730chis Rev	ATCTCAGTGGTGGTGGTGGTGGTGATCCTTATTAGAATCAC GTTTA
pET30a(+)/ *SiL_0731*	SiL_0731 For	GAAATAATTTTGTTTAACTTTAAGAAGGAGATATACATATG GAAAGTAAGTTGAGAGCC
	SiL_0731 Rev	ATCTCAGTGGTGGTGGTGGTGGTGATAAGGATTAGCTAAAT TTCTCATAA
pET30a(+)/ SMV1*gp44chis*	*SMV1gp44*chis For	GAAATAATTTTGTTTAACTTTAAGAAGGAGATATACATATG GTGGCAACGCATAAATTAA
	*SMV1gp44*chis Rev	ATCTCAGTGGTGGTGGTGGTGGTGCCCCTGGTTTTTATTCTC TTTTA

Restriction sites utilized for cloning are underlined.

Supplementary table 2: Source data related to supplementary figure 8.

	**Time (hours)**	**0**	**12**	**24**	**36**	**48**
gp09	**Uninfected**	0.2	0.414	0.992	1.451	1.761
		0.2	0.534	0.959	1.463	1.676
	**SIRV2M (parental virus)**	0.2	0.33	0.387	0.364	0.35
		0.2	0.472	0.325	0.326	0.376
	**Δ*acrIA***	0.2	0.405	0.849	1.35	1.668
		0.2	0.48	0.81	1.261	1.638
	**Δ*acrIIIB1***	0.2	0.427	0.844	1.473	1.709
		0.2	0.478	0.795	1.227	1.648
	**Δ*acrIA*Δ*acrIIIB1***	0.2	0.43	0.843	1.493	1.681
		0.2	0.486	0.863	1.333	1.653
gp10	**Uninfected**	0.2	0.47	0.903	1.437	1.629
		0.2	0.482	0.813	1.229	1.552
	**SIRV2M (parental virus)**	0.2	0.43	0.316	0.322	0.32
		0.2	0.394	0.305	0.328	0.299
	**Δ*acrIA***	0.2	0.407	0.683	1.38	1.593
		0.2	0.407	0.683	1.039	1.593
	**Δ*acrIIIB1***	0.2	0.393	0.833	1.402	1.639
		0.2	0.482	0.753	1.226	1.851
	**Δ*acrIA*Δ*acrIIIB1***	0.2	0.456	0.834	1.497	1.609

		0.2	0.425	0.724	1.11	1.683
gp14	**Uninfected**	0.2	0.448	0.873	1.403	1.669
		0.2	0.482	0.813	1.229	1.552
	**SIRV2M (parental virus)**	0.2	0.391	0.286	0.273	0.292
		0.2	0.353	0.293	0.262	0.246
	**Δ*acrIA***	0.2	0.404	0.86	1.373	1.61
		0.2	0.451	0.857	1.292	1.746
	**Δ*acrIIIB1***	0.2	0.363	0.843	1.365	1.59
		0.2	0.486	0.805	1.326	2.064
	**Δ*acrIA*Δ*acrIIIB1***	0.2	0.367	0.867	1.477	1.643
		0.2	0.444	0.824	1.322	1.94
gp15	**Uninfected**	0.2	0.432	0.866	1.456	1.716
		0.2	0.486	0.805	1.326	2.064
	**SIRV2M (parental virus)**	0.2	0.378	0.286	0.282	0.296
		0.2	0.397	0.341	0.39	0.311
	**Δ*acrIA***	0.2	0.433	0.756	0.952	1.299
		0.2	0.433	0.716	0.899	1.303
	**Δ*acrIIIB1***	0.2	0.463	0.879	1.375	1.672
		0.2	0.501	0.911	1.363	1.795
	**Δ*acrIA*Δ*acrIIIB1***	0.2	0.415	0.866	1.505	1.696
		0.2	0.473	0.871	1.36	1.812
gp16	**Uninfected**	0.2	0.471	0.852	1.288	1.801
		0.2	0.529	0.907	1.43	1.909
	**SIRV2M (parental virus)**	0.2	0.349	0.255	0.278	0.285
		0.2	0.423	0.36	0.349	0.36
	**Δ*acrIA***	0.2	0.395	0.751	1.058	1.65
		0.2	0.485	0.868	1.346	1.728
	**Δ*acrIIIB1***	0.2	0.404	0.812	1.281	1.785
		0.2	0.495	0.916	1.4	1.873
	**Δ*acrIA*Δ*acrIIIB1***	0.2	0.461	0.823	1.207	1.736
		0.2	0.47	0.871	1.333	1.793
gp17	**Uninfected**	0.2	0.499	0.9	1.48	1.706
		0.2	0.437	0.773	1.201	1.757
	**SIRV2M (parental virus)**	0.2	0.342	0.356	0.278	0.306
		0.2	0.336	0.282	0.333	0.291
	**Δ*acrIA***	0.2	0.477	0.873	1.462	1.627
		0.2	0.435	0.689	1.008	1.58
	**Δ*acrIIIB1***	0.2	0.488	0.838	1.42	1.682
		0.2	0.452	0.69	1.065	1.531
	**Δ*acrIA*Δ*acrIIIB1***	0.2	0.416	0.853	1.432	1.667
		0.2	0.443	0.676	1.083	1.638
