# Supplementary material for: Regulatory sequence-based discovery of anti-defense genes in archaeal viruses
Source: Nat Commun. 2024 May 2;15:3699. doi: 10.1038/s41467-024-48074-x (PMC11065993; doi:10.1038/s41467-024-48074-x)
Supplement: Supplementary file 3 — Description of Additional Supplementary Files [file 41467_2024_48074_MOESM3_ESM.pdf]

<u>**Description of Additional Supplementary Files**</u>

Supplementary Data 1. List of all predicted ADGs and the corresponding motif prevalence for the predicted ADGs; ADGs classified into families based on protein sequence similarity; comparison of ADGs identified here to those predicted using DeepAcr and AcrNET.

Supplementary Data 2. RSAT-CRISPR_I-A_Promoter_seq. RSAT analysis of *S. islandicus* LAL14/1 promoters using the consensus sequence GAAATAGAAAGKTTTATATA. Predicted promoters are present upstream of genes which are highly conserved and encode important proteins.

Supplementary Data 3. (1) Multiple sequence alignment of *SMV1* gp44/SiL_0730 homologs. (**2)** Multiple sequence alignment of SiL_0731 homologs.

Supplementary Data 4. DALI output of possible structural matches for AlphaFold predicted structures of *ARV3* gp05, SiRe_2373 (N-terminal) and SiRe_2373 (C-terminal), respectively.

Supplementary Data 5. Information on viral protein families with a high similarity to proteins encoded by Sulfolobales genomes.
