# Supplementary material for: Regulatory sequence-based discovery of anti-defense genes in archaeal viruses
Source: Nat Commun. 2024 May 2;15:3699. doi: 10.1038/s41467-024-48074-x (PMC11065993; doi:10.1038/s41467-024-48074-x)
Supplement: Supplementary file 6 — Supplementary Data 3 [file 41467_2024_48074_MOESM6_ESM.pdf]

```
Reference sequence (1): YP_009008111.1
Identities normalised by aligned length.
Colored by: identity

                     cov    pid   1 [        .         .         .         .         :         .         .         .         .       ] 98
 1 YP_009008111.1 100.0% 100.0%     MKCEDIINSMVQYCAIFNREFEMVA----THKLTDEQKKILERMHNRVDYILETYKEYLDALAEFDRTGVLKIHGKVIYVKENKNQ-----------G
 2 WP_012713850.1  73.5%  67.6%     ----------------------MVI----THKLTDEQKKILERMHSRVDYIFETYREYFDTLAEFDRTGVLKIHGKVLYVRKYENEK-ENEDKYLNLQ
 3 WP_012716295.1  73.5%  67.6%     ----------------------MVI----THKLTDEQKKILERMHSRVDYIFETYREYFDKLAEFDRTGVLKIHGKVLYVRKYENEK-ENEDKYLNLQ
 4 YP_009218527.1  67.5%  75.4%     ----------------------M---------VTDEQKKILERMHSRVDYIVEKYKEYLDALAEFDRTGVLKIHGKVLYVKEYKNQ--GVRG-----S
 5 MCQ4343981.1    73.5%  67.6%     ----------------------MVI----TPKLTDEQKKTLERMHNRVDYIFETYKEYFDALAEFDRTGVLKIHGKVLYVRKYESEK-ENEDKYLNLQ
 6 WP_088922599.1  73.5%  67.1%     ----------------------MVI----THKLTDEQKKILERMQSRIDYIIKAHKEYLDALAEFDRTGVLKIHGKVLYVRKYNNQ--ENEDKRFNLQ
 7 YP_009230287.1  73.5%  71.2%     ----------------------MVV----ANKLTEEQKKILERMHNRVDYIIKAYKEYLDALAEFDRTGVLKIHEKVLYVRKYNNQEN--EPP----K
 8 WP_012953061.1  73.5%  65.7%     ----------------------MVI----AHKLTDEQKKILERMQSRIDYIIKAHKEYLDALAEFDRTGVLKIHGKVLYVRKYNNQ--ENEDKQFNLQ
 9 WP_009991957.1  73.5%  65.7%     ----------------------MVI----AHKLTDEQKKILERMQSRIDYIIKAHKEYLDALAEFDRTGVLKIHGKVLYVRKYNNQ--ENEDKRFNLQ
10 MCG2909053.1    95.2%  55.9%     MAQIFY-KS---FNSIANRELKMVI----TPKLTDEQKKTLERMHNRVDYIFEIYREYFDALAEFDRTGVLKIHGKVLYVRKYESEK-ENEDKYLNLQ
11 PVU74284.1      73.5%  65.7%     ----------------------MVI----AHKLTDEQKKILERMQSRIDYIIKVHKEYLDALAEFDRTGVLKIHGKVLYVRKYNNQ--ENEDKRFNLQ
12 PVU75519.1      73.5%  65.7%     ----------------------MVI----THKLTDEQKKILERMQSRIDYIIKAHKEYLDALAEFDRTGVLKIHGKVLYVRKYNNR--ENEDKRFNLQ
13 WP_012718863.1  95.2%  58.5%     MKIDII-SS---IDVIVNREFKMVV----SHNLTDEQKKILERMRDRVDYIIKTHKEYLDALAEFDRTGVLKIHGKVLYVRKHMGQEEENKDKQLNLQ
14 WP_013776033.1  72.3%  64.3%     ----------------------MVI----THKLTDEQKKILERMQSRIDYIIKAHKEYLDALAEFDRTGVLKIHGKVLYVRKYNQ---ENEDKRFNLQ
15 YP_009226296.1  68.7%  75.4%     ----------------------MV-----EHKLTDEQKKILERMHKRVDYIVDAYKEYLDALAEYDRTGVLKIHGKVLYAKKE--------------K
16 WP_052846491.1  73.5%  65.2%     ----------------------MVV----SHNLTEEQKKILERMQNRINYIIKAHKEYLDALAEFDRTGILKIHGKVLYVRKYKNGQE-NED-----K
17 YP_009362831.1  72.3%  71.0%     ----------------------MTR----VHKLTAEQKKILERMHNRIDYIIKAYKEYLNALAEFDRTGVLKIHGKVIYERKYNDH--EN--------
18 YP_009362552.1  72.3%  71.0%     ----------------------MAR----VHKLSAKQKKIIKRMHNRIDYILEKYKEYLDALAEFDRTGVLKIHGKVIYERKYNDQ--KK--------
19 WP_054845482.1  73.5%  61.1%     ----------------------MVV----ANKVTDEQKKILERMRDRVGYIINAYKEYLDALAEFDRTGVLKIHGKVLYVRKYIEQEEENKNKRLNLQ
20 YP_009362555.1  72.3%  67.7%     ----------------------MTR----VHKLTAEQKKILERMRNRINYIIETYSEYLNALSEFDRTGVLKIHGKVIYERKYNDH--EN--------
21 YP_009362678.1  72.3%  67.7%     ----------------------MTR----VHKLTAEQKKILERMRNRINYIIETYSEYLNALSEFDRTGVLKIHGKVIYERKYNDH--EN--------
22 WP_236753206.1  71.1%  58.2%     ----------------------MAI------TISDEDRKILKRMHERVDYIFSLYKEYFDALAEFDRTGVLKIHGKVLYVREYHVNE-K---NYLD-T
23 WP_229571002.1  71.1%  59.7%     ----------------------MAI------IISDEDKKILKRMHGRVDHILSLYKEYFDALAEFDRTGVLKIHGKVLYVKKYHENK-D---NYLE-T
24 WP_052846537.1  71.1%  56.7%     ----------------------MAI------VISDEDKKVLKRMHGRVDHILSLYKEYFDALAEFDRTGVLKIHGKVLYVRKYHENK-D---NYLE-A
25 WP_054837064.1  73.5%  45.6%     ----------------------MGL----AIAIRDEDKDILKRMHERVDHVLSSHREYFDALKEFDKTGVLKIRGKILYVRRYQETE-D---GNLNLQ
26 WP_184651096.1  66.3%  50.0%     --------------------------------MTDEISEIRNDLYKRAEFVLKTYKKYLDALAEFDRSGVLKVDGKILYVTKREVNK----------G
27 WP_232616535.1  66.3%  42.6%     --------------------------------MTDQISEIRNDLYKRAEFVIKTYKKYLDALAEFDKTGVLKVDGKILYVAKRKTNN-GQSD--F--S
28 WP_156005284.1  73.5%  40.9%     ----------------------MRNKIYNLEKMTEQTSETGKDLYMRAEFVIKTYKKYLDALAEFDRTGILKVDGKILYVAERKANN----------D
29 WP_238527872.1  48.2%  54.0%     -----------------------------------------------MGHSLETYREYFDALAEFDRTGVLKIHGKVLYVRKYENEK-ENEDKYLNLQ
30 WP_229571697.1  66.3%  46.4%     --------------------------------MTDEISEIRNDLYKRAEFVIKAYKKYLDALAEFDKTGVLKVDGKVLYVAKREVNK----------D
31 WP_016729867.1  72.3%  38.7%     ----------------------MYK-----RKMTEQVSEIQKDLHKRAEFVIKAYKKYFDALAEFDKTGILKVNGEVLYVSKRDSNK----------D
32 WP_009074432.1  73.5%  41.2%     ----------------------MVMLE-FDTSLTGEEVSLLEALNRRADFVLDEYKDYFKALAEFEKTGVLKLKGKEIYVSKRIRPM-K-S---L--P
33 WP_170254143.1  68.7%  41.9%     ----------------------MT----V---NQVEIEEIRRDLYKRAEFVIKAYKKYLDALAEFDRTGILKIDGEVLYVAKYKEN-----------K
34 ACP37521.1      72.3%  37.1%     ----------------------MYK-----RKMTEQVSEIQKDLRKRAEFVIKAYKKYFDALAEFDKTGILKVNGEVLYVSKRDSNK----------D
35 ACP54664.1      72.3%  37.1%     ----------------------MYK-----RKMTEQVSEIQKDLRKRAEFVIKAYKKYFDALAEFDKTGILKVNGEVLYVSKRDSNK----------D
36 ACR41344.1      72.3%  37.1%     ----------------------MYK-----RKMTEQVSEIQKDLRKRAEFVIKAYKKYFDALAEFDKTGILKVNGEVLYVSKRDSNK----------D
37 ADX81997.1      72.3%  37.1%     ----------------------MYK-----RKMTEQVSEIQKDLRKRAEFVIKAYKKYFDALAEFDKTGILKVNGEVLYVSKRDSNK----------D
38 ADX84776.1      72.3%  37.1%     ----------------------MYK-----RKMTEQVSEIQKDLRKRAEFVIKAYKKYFDALAEFDKTGILKVNGEVLYVSKRDSNK----------D
39 WP_236752582.1  68.7%  41.5%     ----------------------MV----V---SQSEIEEIRKDLYKRAEFVIKAYKEDLDALAEFDRTGVLKLNGKVLYVAKFNDDK-K-D------Q
40 WP_012710794.1  72.3%  37.1%     ----------------------MYK-----RKMTEQVSEIQKDLRKRAEFVIKAYKKYFDALAEFDKTGILKVNGEVLYVSKRDSNK----------D
   consensus/100%                   ...............................................hta.hp.apc.hptLtEa-+oGlLKlptc.lY.tc................
   consensus/90%                    ......................M.........hotp.pcl.cch.pRhpalhpha+cYhDALAEFD+TGlLKlpGcllYst+hptp............
   consensus/80%                    ......................Mh........hT-p.pcIhcchppRh-allcsaKcYhDALAEFD+TGlLKlcGKVLYVpKhpsp...........p
   consensus/70%                    ......................Mhh......phT-E.pcIhcchppRs-allcsYK-YhDALAEFDRTGVLKlcGKVLYVpKhpsp...........p
```

MView 1.63, Copyright © 1997-2018 Nigel P. Brown

```
Reference sequence (1): WP_010979162.1
Identities normalised by aligned length.
Colored by: identity


                         cov    pid   1 [         .         .         .         .         :         .         .         . 80
 1 WP_010979162.1 100.0% 100.0%       ----MENKLMQILEGLLNEFEKWRSRESD-----RVPTIIQIHDSITRNDP-NTERGIVNLDTIGITIY---SAIENLSL
 2 WP_232049044.1  75.7%  78.3%       ----------------------------------------------MKRDT-NTEPGIINVDVIGIAIY---SAIEDLIN
 3 WP_012718862.1 100.0%  80.3%       ----MESKLMQILEGLLNEFEDWRSGKSN-----IEPTIIKIHDSIIRSDP-NTERGIINLDVIGIAIY---SAIEDLSN
 4 WP_012953062.1 100.0%  81.6%       ----MESKLMQILEGLLNEFEEWRSRKGD-----IEPTIIKIHYSIIRSDP-NTELGIINLDVIGIAIY---SAIEDLNN
 5 PVU75520.1     100.0%  80.3%       ----MESKLMQILAGLLNEFEEWRSRKGD-----IEPTIIKIHYSIIRSDP-NTEQGIINLDVIGIAIY---SAIEDLNN
 6 WP_048054638.1  97.4%  81.1%       ----MESKLMQILAGLLNEFEEWRRGESD-----IEPTIIKIHYSIIRSDP-NTEQGIINLDVIGIAIY---SAIEDLNN
 7 WP_009991959.1 100.0%  81.6%       ----MESKLMQILEGLLNEFEEWRRGESD-----IEPTIIKIHYSIIRSDP-NTEQGIINLDVIGIAIY---SAIEDLNN
 8 PVU74283.1     100.0%  81.6%       ----MESKLMQILAGLLNEFEEWRRGESD-----IEPTIIKIHYSIIRSDP-NTEQGIINLDVIGIAIY---SAIEDLNN
 9 WP_088922621.1 100.0%  80.3%       ----MESKLMQILAGLLNEFEEWRRGESD-----IEPTIIKIHYSIIRSDP-NTEQGIINLDVIGIAIY---SAIEDLNN
10 WP_054837063.1 100.0%  56.5%       MSNLLEQKLLEIFNALLKEFNIWRKGESQ-----EEPLIINIHDKVVANDP-TSAQGIMNRDNIGLTIY---SAITDLMR
11 WP_229571003.1 100.0%  56.3%       ---MLEEKLLKILEVLLKEFEAWIRGKSE-----EKPLIIEIHDKLIANNT-YSEGGVINLDDIGIAIY---SAIEDLMR
12 WP_052846538.1 100.0%  54.7%       MNILLEEKLLKILNALLKEFEAWIRGESE-----EKPLIIEIHDKLIANDT-YSEGGVINLDDIGIAIY---SSIEDLMR
13 WP_229571468.1 100.0%  58.2%       MSILLEETLLKILRLILREFEAWNIGESK-----EKPLIIKIHDKVIASNA-ESEQGIINSDNIGIAIY---SAIEDLNQ
14 WP_016731964.1 100.0%  58.9%       MSILLEETLLKILRLILREFEAWSSGESK-----EKPLIIKIHDKVIANNA-ESEQGIINSDNIGIAIY---SAIEDLNQ
15 MCQ4343980.1   100.0%  64.9%       ----MESKLMQILAGLLNEFEEWRRGDSD-----KEPLIIEIHEKVVSNDP-YTEQGVINLDDVSIAIY---SAIEDLMR
16 WP_238526128.1  78.9%  63.1%       -----------------------------------------MHDKVVSNDP-YTEQGVINLGDVSIAIY---SAIEDLMR
17 WP_238526249.1  73.0%  60.2%       --------------------------------------------------M-HGLSETVVNGDVSIAIY---SAIEDLMR
18 WP_238527871.1  69.7%  62.0%       --------------------------------------------------------MDFIKGDVSIAIY---SAIEDLMR
19 WP_236752583.1  90.1%  34.8%       ----MESKFQTILNSLDKDFIIWLNQQV-----------------VNE-DP-SSTFGLVNPDNIDGAIY---SAIYDFEE
20 MQL56197.1      64.5%  39.6%       ---------------------------------------------------------------MDGAIY---SAIFDLEE
21 WP_231136444.1  59.2%  37.8%       --------------------------------------------------------------------------MFDLEE
22 WP_016729866.1  89.5%  34.6%       ----MESKLRAIITSLSKTFIIWLNDRV-----------------LKEEDP-SLTSIVINEGNIEGAIY---SAILDFEI
23 WP_012710793.1  89.5%  34.0%       ----MESKLRAIITSLSKTFIIWLNDRV-----------------LKEEDP-SLTSIVINEGNIEGAIY---SALLDFEI
24 WP_014512183.1  89.5%  33.3%       ----MESKLRAIITSLSKTFIIWLNDRV-----------------LKEEDP-SLTSIVINEGNIEGAIY---SALLDFEI
25 WP_012735676.1  89.5%  33.3%       ----MESKLRAIITSLSKTFIIWLNDRV-----------------LKEEDP-SLTSIVINEGNIEGAIY---SALLDFEI
26 WP_012718553.1  89.5%  32.7%       ----MESKLRAIITSLSKTFIIWLNDRV-----------------LKEEDP-SLTSIVINEGNIEGAIY---SALLDFEI
27 WP_052846531.1  89.5%  30.2%       ----MENKFRLIVTSLDKSFITWLNDRV-----------------LKEEDP-SLTSTVINEGNIEGAIY---SAVMDFEI
28 WP_156013372.1  89.5%  32.1%       ----MGNKFQLILASLDKSFIIWLNERV-----------------LKEEDP-SLTSIVINEGNIEGAVY---SAMLDFEI
29 WP_229571696.1  89.5%  31.4%       ----MENKFQMILASLDKNFIIWLNERV-----------------LKEDDP-SLTSIVINEGNIEGAIY---SAILDFEI
30 MBU4491207.1    67.1%  17.4%       -----------------------------------------------EETGGN--KGVLNFGNLKASVSRPFAAFGDQEL
31 MCL4339950.1    77.6%  18.5%       ---------------------------MRYQYL-TREQVVAIHEEVIMKYGGS--EGIISESNIELAISRPNSVLFGYEP
32 MBI2676267.1    71.1%  14.4%       ---------------------------MNSEYIIDKNQIRLIN----EMYGGY----LRSDAEIETAL--------YLGK
33 MBI5061864.1    71.1%  18.0%       ---------------------------MNSEYIITKEQIKWIN----EQYGGS----LRTDAEIETAL--------DLGK
34 MCK4714356.1    71.1%  15.8%       ---------------------------MNSEYIIPKEQIKAIN----ERYGGS----LRTDAEIETAL--------SMGR
35 WP_052368754.1  77.0%  18.9%       ----------------------MVEAEKDILIP-STDDIITIN----QKLGGT----VLNRGIIDFIIAKIEAKVPKKDY
36 MBU0586829.1    75.7%  18.2%       ----------------------------MLKYL-SSLQIISIHDRIIEEFSKE--KGQMNIGNLEATLERVMD--YKGND
37 MBN1133826.1    75.0%  18.8%       -----------------------------MTEI-STKSIIDIHNRIIKEFGGDSTPGILSEGNIDFIVF-------EVDR
38 MBI4894851.1    73.7%  19.7%       -----------------------------MGDL-TVGDVVEINRSIVDRFGVT--FGVLNMGNLDFVVE-------SAMH
39 MCL5239032.1    72.4%  19.7%       ---------------------------MEILYP-TTEEIVEIN----KKIGCD--GKFINQGNLDFTLD-------KARN
40 MBI2547095.1    73.0%  21.1%       -----------------------------MKYL-TTQEILHIHDDIIKETGGH--GGILSYGNLDFIVN-------QMKI
41 MBI2543485.1    75.0%  19.9%       ---------------------------MKVVYL-TIEEVVKIHEDMIKETGGH--PGIISYGNLDFTVN-------QAKI
42 MBI2085413.1    73.0%  23.5%       ------------------------------MKI-VIRAIIQLHDKIIRKSGGH--AGMINVGNLDFTVA-------QANY
43 MBN2488821.1    66.4%  18.3%       ---------------------------------------------MIENYGGA--KGTLYSATLDYIVY-------EINK
44 NJD52472.1      85.5%  20.9%       ---------MTIYDS--RVFKQVRSKGNQMAEL-TVRKIIEIHDEIIQKYGGT--SGVLSEATLEMLVY-------KVNR
45 MBU4220955.1    73.7%  23.0%       ------------------------------MFL-TARQIIEIHDTILKKYGGT--GGVLNEGTLELLVY-------KTSR
46 MCG7849420.1    73.7%  22.9%       -----------------------------MEEF-TVEQIIEIHDRIIDKYGGT--GRMLNTGTLELLVY-------KLNR
   consensus/100%                     ................................................................................
   consensus/90%                      ..................................................s......hhs.s.lthhl........ph..
   consensus/80%                      ...............................................tp.s.p...hlls.sslthsl........cht.
   consensus/70%                      .............................................lhpp.s.p..thllN.sslthsla.......chp.

                         cov    pid  81         .         1         .         .         .         .         :         . 160
 1 WP_010979162.1 100.0% 100.0%       YHDISRSLAVLTYRLITSHPFVDANKRTAFVLLLDILYELFDK--E------IPQDLEEELI--KTLAEVADNPPEEDEY
 2 WP_232049044.1  75.7%  78.3%       YHDISRSLAVLTYHLITSHPFVDANKRTTFVLLLNILYELYDK--E------VPQDLEEELI--KTLVEVADNPPKEDEY
 3 WP_012718862.1 100.0%  80.3%       YHDISRSLAVLTYHLITSHPFVDANKRTAYILLLNILYELSVK--E------IQQNLEEELI--KTLVEVADNPPEEDEY
 4 WP_012953062.1 100.0%  81.6%       YNDISRSLAVLTYHLITSHPFVDANKRTAFVLLLNILYELSNK--E------IPQDLEEELI--KTLVEVADNPPEEDEY
 5 PVU75520.1     100.0%  80.3%       YNDSSRSLAVLTYHLITSHPFVDGNKRTAFVLLLNILYELFNK--E------IPQDLEEELI--NTLVEVADNPPEEDEY
 6 WP_048054638.1  97.4%  81.1%       YNDISRSLAVLTYHLITSHPFVDGNKRTAFVLLLNILYELFNK--E------IPQDLEEELI--KTLVEVADNPPEEDEY
 7 WP_009991959.1 100.0%  81.6%       YNDISRSLAVLTYHLITSHPFVDGNKRTAFVLLLNILYELFNK--E------IPQDLEEELI--NTLVEVADNPPEEDEY
 8 PVU74283.1     100.0%  81.6%       YNDISRSLAVLTYHLITSHPFVDGNKRTAFVLLLNILYELFNK--E------IPQDLEEELI--KTLVEVADNPPEEDEY
 9 WP_088922621.1 100.0%  80.3%       YNDISRSLAVLTYHLITSHPFVDGNKRTAFVLLLNILYELFNK--E------IPQDLEKELI--KTLVEVADDPPEEDEY
10 WP_054837063.1 100.0%  56.5%       NYDISRSLAVLTYHLVVSHPFIDGNKRTALGLLLNILYELFEDKME------IPQDLEDQLI--RVLIEISDYPPEEDED
11 WP_229571003.1 100.0%  56.3%       NHDVSRSLAVLTYHLVISHPFVDGNKRTTLGFLLEILHDLFEDKIE------IPQDLVDRLM--QTLVEIADNPPEEDEV
12 WP_052846538.1 100.0%  54.7%       NHDVSRSLAVLTYHLVVSHPFVDGNKRTTLGFLLNILHTLFEDEIE------IPQDVVDTLM--QTLVEIADNPPEEDEM
13 WP_229571468.1 100.0%  58.2%       YHDVSRSLAVLIYHLIVSHPFVDGNKRTALGLLLHILHEVCNDIIT------IPPALMELLL--KTLAEVADYSPEEDEY
14 WP_016731964.1 100.0%  58.9%       NHDVSRSLAVLIYHLIVSHPFVDGNKRTALGLLLHILHEMCNDIIT------IPPASMELLL--KTLAEVADYSPEEDEY
15 MCQ4343980.1   100.0%  64.9%       SNDISRSLAVLTYHLITSHPFVDGNKRTALGLLLHILHELFNDNVS------ISPDLLDLLI--KTLTEVADVQPEEDEH
16 WP_238526128.1  78.9%  63.1%       NNNISRSLAVLTYHLITSHPFVDGNKRTTLGLLLHILHELFNDKIS------ILPDLLDLLI--KTLTEVADNPPEEDEH
17 WP_238526249.1  73.0%  60.2%       NNNISRSLAVLTYHLITSHPFVDGNKRTALGLLLHILHELFNDKIS------ILPDLLDLLI--KTLTDVADNPPEEDEH
18 WP_238527871.1  69.7%  62.0%       NNNISRSLAVLTYHLITSHPFVDGNKRTALGLLLHILHELFNDKIS------ILPDLLDLLI--KTLTDVADNPPEEDEH
19 WP_236752583.1  90.1%  34.8%       NHNISRSLAVLIHHIASGHPFADGNKRTSYALLLTIISRLSDKNILDLRQIIWNSGLREIIL--TTLAEISSD--EDEEK
20 MQL56197.1      64.5%  39.6%       NHDISRSLAVLIHHIASGHPFADGNKRTSYALLLSILSKLYEKDIL------LDSKLAKKLT--ITIAEISGE-SEDEEK
21 WP_231136444.1  59.2%  37.8%       NHDISRSLAVLIHHIASGHPFADGNKRTSYALLLSILSKLYEKDIL------LDSKLAKKLT--ITIAEISGE-SEDEEK
22 WP_016729866.1  89.5%  34.6%       NHSISRSLAVLIHRLISGHPFADGNKRTATALLLTILSKLYERDII------IEDRLMKSLI--TVIAKIANE-T---EN
23 WP_012710793.1  89.5%  34.0%       NHSISRSLAVLIHRLISGHPFADGNKRTATALLLTILSKLYERDII------IEDRLMKSLI--TVIAKIANE-T---EN
24 WP_014512183.1  89.5%  33.3%       NHSISRSLAVLIHHLISGHPFADGNKRTATALLLTILSKLYERDIT------IEDRLMKSLI--TVIAKIANE-T---EN
25 WP_012735676.1  89.5%  33.3%       NHSISRSLAVLIHHLISGHPFADGNKRTATALLLTILSKLYERDII------IEDRLMKSLI--TVIAKIANE-T---EN
26 WP_012718553.1  89.5%  32.7%       NHSISRSLAVLIHHLISGHPFADGNKRTATALLLTIISKLYERDII------IEDRLMKSLI--TVIAKIANE-T---EN
27 WP_052846531.1  89.5%  30.2%       NHNISRSLAVLIHHLVVGHPFADGNKRTATTLLITLISKLYERDVV------LEENLMNSLI--TIIAEISNE-S---EN
28 WP_156013372.1  89.5%  32.1%       NHNISRSLAVLIHHLITGHPFADGNKRTATALLLTILSKLYERDII------LEETLMNSLI--TIIAKISKE-P---EN
29 WP_229571696.1  89.5%  31.4%       NRSISRSLAVLVHHLVIGHPFADGNKRTVTALLITILSKLYERDIA------IDESLMNSLI--TTIAKISNE-P---EN
30 MBU4491207.1    67.1%  17.4%       FPDLTAKVAAQIHAISSYHPFVDGNKRTALVAADVCLRLNGYCLTN---------SDDVES---FFWS-IARG-----EQ
31 MCL4339950.1    77.6%  18.5%       FRTLTEKAAVLFHDIDKLHPFVDGNKRTAYTAADIFLRLNGKKIQA---------STEAAV---FISLETAKC-----SA
32 MBI2676267.1    71.1%  14.4%       GKSVYRKIACLWKAILVGHSFTDGNKRTALIVALALLEKSGIRLGD---------KSKENMV--SEVTKIAKE-----NI
```

```
 33 MBI5061864.1     71.1%  18.0%      GRNVYRKIAYLLKAILVGHPFTDGNKRTALMVSLAILESCEIKIAG---------KQKENLV--EEILKVAQE-----NI
 34 MCK4714356.1     71.1%  15.8%      GRNVYRKIAALWRAILVGHPFTDGNKRTALMVALYILESCDIRIPE---------PRKESIV--REITKIARE-----NI
 35 WP_052368754.1   77.0%  18.9%      KRQIATIAAVFWFEIIQGHPFVDGNKRTGTEIMKLFLKKNGFKLNT---------PLAGLV---YISLKIANN-----EI
 36 MBU0586829.1     75.7%  18.2%      SEALFWKATIMLERIILGHPFIDGNKRTGYEATKIFLLANGYRLII---------KEEEVI---AMLIAIAQN-----KK
 37 MBN1133826.1     75.0%  18.8%      KKDLFEKGAFMLCNLITSHPFVDGNKRTAFQTTNLVFRNNGYFIFA---------KDDDVE---DCLLKIASY-----KC
 38 MBI4894851.1     73.7%  19.7%      SQDVFRKAAELMYGIARGHPFMDGNKRTAFEAAFLTLDSFGIALKV---------EPKEAE--DFMLRIVSSE-----SL
 39 MCL5239032.1     72.4%  19.7%      AKALTKKAAILLEGIVTGHPFVDGNKRTAFVAMELFLKKNGKELEH---------TKSDEHLMERVLYDIAEN-----RI
 40 MBI2547095.1     73.0%  21.1%      PKTIERSAIVLFYGILTSHPFVDGNKRTGLESMKTFLYLNGKRFVA---------EDNDIW---SKVHDTSEG-----KL
 41 MBI2543485.1     75.0%  19.9%      PKNIERIATTLFYGILTSHPFIDGNKRTAISVLETFLEENDARLVA---------KNEELW---NIVHIVSEG-----KL
 42 MBI2085413.1     73.0%  23.5%      VGNLTKKLATLLFGIVARHPFVDGNKRTALVVVEAILRTAGKRLTA---------GEKDLW---VLLNKISIG-----EM
 43 MBN2488821.1     66.4%  18.3%      NNDVYRNAALVVHKIVTGHPFMDGNKRTALGIAHIYLESKGLYITA---------SKKDKL---ELLLKTASY-----KV
 44 NJD52472.1       85.5%  20.9%      EHNVLRQSSMVLHMITSQHPFFDGNKRTALVTAEKMLYDEGYYIHA---------EAEKKV---DFMIKIAEY-----KC
 45 MBU4220955.1     73.7%  23.0%      EKDIFRKAALILHTIAAQHPFFDGNKRTALAAAENTLGEAGYYLHA---------DDEEIV---CLMQKIAEY-----KC
 46 MCG7849420.1     73.7%  22.9%      EKDIFRQAALFLHAIAAQHPFFDGNKRTAFAASENVLQEAGYYLNA---------ETNEIV---NLMQKIAEY-----KC
    consensus/100%                     ..t........h..l...HsFhDuNKRTsh.....hh................................hu.......p.
    consensus/90%                      .psl.cphAhhhhtlh.tHPFhDGNKRTsh.hh..hL.p................t..p.h....hhhclup......p.
    consensus/80%                      .psl.RphAlLhatllsuHPFsDGNKRTuhshhhthL.p.h.c............p..c.l...phlhclupt.....c.
    consensus/70%                      .psloRplAlLhacllsuHPFsDGNKRTAhslhhplLtchhtc..t.........ph.c.lh..phlhclApp.....ch

                         cov    pid 161       .         .      ] 187
  1 WP_010979162.1 100.0% 100.0%      AINKIRETIRQIIEG------------
  2 WP_232049044.1  75.7%  78.3%      TINKIRKIIQKIIED------------
  3 WP_012718862.1 100.0%  80.3%      AISKIRKIIQKIIED------------
  4 WP_012953062.1 100.0%  81.6%      AINKIRKIIRKFIED------------
  5 PVU75520.1     100.0%  80.3%      AINKIRKIIRKIIED------------
  6 WP_048054638.1  97.4%  81.1%      AINKIRKIIED----------------
  7 WP_009991959.1 100.0%  81.6%      AINKIRKIIRKIIED------------
  8 PVU74283.1     100.0%  81.6%      AINKIRKIIRKIIED------------
  9 WP_088922621.1 100.0%  80.3%      AINKIRKIIRKIIED------------
 10 WP_054837063.1 100.0%  56.5%      GINRIRNIIEDIIHGILF---------
 11 WP_229571003.1 100.0%  56.3%      AISRIRSIIRDLIPVNQD---------
 12 WP_052846538.1 100.0%  54.7%      AINRVRSIIRGLIPVNQD---------
 13 WP_229571468.1 100.0%  58.2%      AINKIREIIMQIIRD------------
 14 WP_016731964.1 100.0%  58.9%      AINKIREIIRQIIRD------------
 15 MCQ4343980.1   100.0%  64.9%      AINKIRGIIQRIIGD------------
 16 WP_238526128.1  78.9%  63.1%      AINKIRGIIQRIIGD------------
 17 WP_238526249.1  73.0%  60.2%      AINKIRGIIQRIIGD------------
 18 WP_238527871.1  69.7%  62.0%      AINKIRGIIQRIIGD------------
 19 WP_236752583.1  90.1%  34.8%      SINTLQEVIEKVIDLSCRH--------
 20 MQL56197.1      64.5%  39.6%      DIRKLQKIIEEIMSTYS-PYT------
 21 WP_231136444.1  59.2%  37.8%      DIRKLQKIIEEIMSTYS-PYT------
 22 WP_016729866.1  89.5%  34.6%      EIRELQEIIGEIMRNLANPY-------
 23 WP_012710793.1  89.5%  34.0%      EIRELQEIIGEIMRNLANPY-------
 24 WP_014512183.1  89.5%  33.3%      EIRELQEIIGEIMRNLANPY-------
 25 WP_012735676.1  89.5%  33.3%      EIRELQEIIGEIMRNLANPY-------
 26 WP_012718553.1  89.5%  32.7%      EIRELQEIIGEIMRNLANPY-------
 27 WP_052846531.1  89.5%  30.2%      DIDKLQKIIEEIMKNLTVHY-------
 28 WP_156013372.1  89.5%  32.1%      DILDLQKIIEEIMKRLANPY-------
 29 WP_229571696.1  89.5%  31.4%      DVDELQRIIEEIMKRLTNPY-------
 30 MBU4491207.1    67.1%  17.4%      SVEDIEAWVKSQTKPCQTKPLINQNRR
 31 MCL4339950.1    77.6%  18.5%      DILQVTDWINKHTSKLSKLDSP-----
 32 MBI2676267.1    71.1%  14.4%      TDVSRIERLVRYAATGN----------
 33 MBI5061864.1    71.1%  18.0%      TDVNRIERLVRYAITGN----------
 34 MCK4714356.1    71.1%  15.8%      TDINRIERLVRYAATEN----------
 35 WP_052368754.1  77.0%  18.9%      SYSELINWLYRRLENGNLY--------
 36 MBU0586829.1    75.7%  18.2%      NRFSIKAWLTKHANLRAQPP-------
 37 MBN1133826.1    75.0%  18.8%      TEKEVKSWLKKRARKVKNTIQLQFS--
 38 MBI4894851.1    73.7%  19.7%      TVREVEMWIRRHGDWYE----------
 39 MCL5239032.1    72.4%  19.7%      TEESLQEIISELIK-------------
 40 MBI2547095.1    73.0%  21.1%      KFEEIINWIKKSVK-------------
 41 MBI2543485.1    75.0%  19.9%      KFEEVVKWIKEVVKW------------
 42 MBI2085413.1    73.0%  23.5%      NVKQIAEWLSKNIA-------------
 43 MBN2488821.1    66.4%  18.3%      NQKEIEKWLQENTRKL-----------
 44 NJD52472.1      85.5%  20.9%      SVKTIEKWVKKSIRELHPG--------
 45 MBU4220955.1    73.7%  23.0%      TVKTIEKWVREKAKATSLPVS------
 46 MCG7849420.1    73.7%  22.9%      SVQKIEKWVREKAAATSGPIS------
    consensus/100%                    t..ph..hl.t................
    consensus/90%                     t..pl.thl.p.ht.............
    consensus/80%                     shpplpphltchht.............
    consensus/70%                     slpclpchIpchhpt............
```

MView 1.63, Copyright © 1997-2018 Nigel P. Brown
