# Supplementary material for: Regulatory sequence-based discovery of anti-defense genes in archaeal viruses
Source: Nat Commun. 2024 May 2;15:3699. doi: 10.1038/s41467-024-48074-x (PMC11065993; doi:10.1038/s41467-024-48074-x)
Supplement: Supplementary file 9 — Reporting Summary [file 41467_2024_48074_MOESM9_ESM.pdf]

nature portfolio

Corresponding author(s): Yuvaraj Bhoobalan-Chitty, Xu Peng

Last updated by author(s): Mar 29, 2024

Reporting Summary

Nature Portfolio wishes to improve the reproducibility of the work that we publish. This form provides structure for consistency and transparency in reporting. For further information on Nature Portfolio policies, see our Editorial Policies and the Editorial Policy Checklist.

Statistics

For all statistical analyses, confirm that the following items are present in the figure legend, table legend, main text, or Methods section.

n/a	Confirmed	
☐	☒	The exact sample size (*n*) for each experimental group/condition, given as a discrete number and unit of measurement
☐	☒	A statement on whether measurements were taken from distinct samples or whether the same sample was measured repeatedly
☐	☒	The statistical test(s) used AND whether they are one- or two-sided<br>*Only common tests should be described solely by name; describe more complex techniques in the Methods section.*
☒	☐	A description of all covariates tested
☒	☐	A description of any assumptions or corrections, such as tests of normality and adjustment for multiple comparisons
☐	☒	A full description of the statistical parameters including central tendency (e.g. means) or other basic estimates (e.g. regression coefficient) AND variation (e.g. standard deviation) or associated estimates of uncertainty (e.g. confidence intervals)
☐	☒	For null hypothesis testing, the test statistic (e.g. *F*, *t*, *r*) with confidence intervals, effect sizes, degrees of freedom and *P* value noted<br>*Give P values as exact values whenever suitable.*
☒	☐	For Bayesian analysis, information on the choice of priors and Markov chain Monte Carlo settings
☒	☐	For hierarchical and complex designs, identification of the appropriate level for tests and full reporting of outcomes
☒	☐	Estimates of effect sizes (e.g. Cohen's *d*, Pearson's *r*), indicating how they were calculated

Our web collection on statistics for biologists contains articles on many of the points above.

Software and code

Policy information about availability of computer code

Data collection	Viral genomes (Sulfolobales) were downloaded from Genbank and Sulfolobales genomes from Refseq databases in August 2022.
Data analysis	PSI-BLAST was used to compare the viral and Sulfolobales proteins.<br>MEME suite (version 5.5.0 or later) was used for motif identification through its website (http://meme-suite.org).<br>RSAT was used to scan the genomes using the motif-matrix generated using MEME suite (http://rsat.sb-roscoff.fr/matrix-scan_form.cgi).<br>PSI-BLAST to determine protein homology. HHpred, DALI server and Alphafold2 were utilized to determine sequence, secondary structure homology or for prediction of structure.<br>GraphPad Prism version 10.0.1 (or later).<br>pyGenomeViz was used to generate figure 3B, visualizing the genomic features of the T6 transcript of SSV viruses.

For manuscripts utilizing custom algorithms or software that are central to the research but not yet described in published literature, software must be made available to editors and reviewers. We strongly encourage code deposition in a community repository (e.g. GitHub). See the Nature Portfolio guidelines for submitting code & software for further information.

Data

Policy information about availability of data

All manuscripts must include a data availability statement. This statement should provide the following information, where applicable:

- Accession codes, unique identifiers, or web links for publicly available datasets
- A description of any restrictions on data availability
- For clinical datasets or third party data, please ensure that the statement adheres to our policy

The authors declare that the data supporting the findings of this study are available within the paper and its supplementary information files. Sulfolobales viral genomes downloaded from GenBank and Sulfolobus genomes from RefSeq database, are openly accessible from NCBI (https://ncbi.nlm.nih.gov/). The accession IDs of virus genomes and proteins used in the identification ADGs are listed in Supplementary Data 1. Source data are provided with this paper. Additional information can be obtained from the corresponding authors upon request.

Research involving human participants, their data, or biological material

Policy information about studies with human participants or human data. See also policy information about sex, gender (identity/presentation), and sexual orientation and race, ethnicity and racism.

Reporting on sex and gender	N/A
Reporting on race, ethnicity, or other socially relevant groupings	N/A
Population characteristics	N/A
Recruitment	N/A
Ethics oversight	N/A

Note that full information on the approval of the study protocol must also be provided in the manuscript.

Field-specific reporting

Please select the one below that is the best fit for your research. If you are not sure, read the appropriate sections before making your selection.

☒ Life sciences ☐ Behavioural & social sciences ☐ Ecological, evolutionary & environmental sciences

For a reference copy of the document with all sections, see nature.com/documents/nr-reporting-summary-flat.pdf

Life sciences study design

All studies must disclose on these points even when the disclosure is negative.

Sample size	The growth curves (figure 4B), virus titration (figure 4C) and transformation assay (figure 5D) are represented by three or more biological replicates. The choice of at least three biological replicates is based on other similar studies and is a standard practice.
Data exclusions	No data was excluded.
Replication	Images shown in figure 4D (spot assay) and figure 5E (pulldown) were derived from a single experiment but their validity was confirmed upon repetition. The spot assay was replicated five times and the pulldown repeated twice (in both cases the results were in agreement to the images shown in the figures).
Randomization	Sulfolobus transformants carrying plasmid encoding different sulfolobus virus or sulfolobus genes were selected randomly. None of the other samples were allocated into experimental groups, hence, randomization is not relevant to this study.
Blinding	Blinding is not applicable in this study. All data obtained correspond to a specific genotype of Sulfolobus islandicus or the virus SIRV2 and its mutants. Also, appropriate controls and replicates were utilized in every experiment, hence there is no potential for bias.

Reporting for specific materials, systems and methods

We require information from authors about some types of materials, experimental systems and methods used in many studies. Here, indicate whether each material, system or method listed is relevant to your study. If you are not sure if a list item applies to your research, read the appropriate section before selecting a response.

Materials & experimental systems

n/a	Involved in the study
☒	☐ Antibodies
☒	☐ Eukaryotic cell lines
☒	☐ Palaeontology and archaeology
☒	☐ Animals and other organisms
☒	☐ Clinical data
☒	☐ Dual use research of concern
☒	☐ Plants

Methods

n/a	Involved in the study
☒	☐ ChIP-seq
☒	☐ Flow cytometry
☒	☐ MRI-based neuroimaging

Plants

Seed stocks	N/A
Novel plant genotypes	N/A
Authentication	N/A
